# Design and Fabrication of an Ag Ultrathin Layer-Based Transparent Band Tunable Conductor and Its Thermal Stability

**DOI:** 10.3390/nano13142108

**Published:** 2023-07-19

**Authors:** Er-Tao Hu, Hongzhi Zhao, Min Wang, Jing Wang, Qing-Yuan Cai, Kehan Yu, Wei Wei

**Affiliations:** 1 College of Electronic and Optical Engineering, Nanjing University of Posts and Telecommunications, Nanjing 210023, China; iamethu@njupt.edu.cn (E.-T.H.); 15062279005@163.com (H.Z.); m18014487392@163.com (M.W.); kehanyu@njupt.edu.cn (K.Y.); weiwei@njupt.edu.cn (W.W.); 2 Department of Basic Education, Tongda College of Nanjing University of Posts and Telecommunications, Yangzhou 225127, China; wangjing@nytdc.edu.cn; 3 Shanghai Institute of Technical Physics, Chinese Academy of Sciences, Shanghai 200083, China

**Keywords:** transparent conductor, TiO_2_/Ag/AZO, optical transmittance, sheet resistance, annealing

## Abstract

Transparent conductors (TC) have been widely applied in a wide range of optoelectronic devices. Nevertheless, different transparent spectral bands are always needed for particular applications. In this work, indium tin oxide (ITO)-free TCs with tunable transparent bands based on the film structure of TiO_2_/Ag/AZO (Al-doped ZnO) were designed by the transfer matrix method and deposited by magnetron sputtering. The transparent spectra and figure-of-merit (FOM) were effectively adjusted by precisely controlling the Ag layer’s thickness. The fabricated as-deposited samples exhibited an average optical transmittance larger than 88.3% (400–700 nm), a sheet resistance lower than 7.7 Ω.sq^−1^, a low surface roughness of about 1.4 nm, and mechanical stability upon 1000 bending cycles. Moreover, the samples were able to hold optical and electrical properties after annealing at 300 °C for 60 min, but failed at 400 °C even for 30 min.

## 1. Introduction

Indium tin oxide (ITO), as a wide bandgap transparent conductor (TC), has been widely applied in a wide range of optoelectronic devices, such as displays, light-emitting diodes (LEDs), and photovoltaic cells (PVs) [1,2,3,4]. However, with the current explosive development of bendable and wearable systems, TCs with high flexibility become essential in flexible optoelectronic devices, which significantly hinders the further applications of ITO because of its poor mechanical flexibility and high annealing temperature for low resistivity. Hence, intensive efforts have been devoted to finding other alternatives, such as Al-doped ZnO (AZO), carbon nanotubes (CNTs), graphene, metal nanowires, metal mesh patterns, and conducting polymers [5,6,7,8,9,10,11,12]. However, these alternatives have disadvantages, for example, high mass-production cost, significant surface roughness, and high optical daze caused by optical scattering, all of which impede their large-area industrial applications [1,2].

Dielectric–metal–dielectric (DMD) film, an ultrathin metal layer sandwiched between two transparent dielectrics, has been identified as a promising TC due to its high optical transparency and electrical conductivity, excellent flexibility, facile fabrication methods, and compatibility with various substrates [1,13,14,15]. Among various DMD-based TCs, dielectric materials such as TiO_2_, Ta_2_O_5_, ITO, MoO_3_, WO_3_, and ZnO-based oxide have been employed to serve as antireflection layers, while Ag has been commonly used as the metal layer owing to its low optical absorption in the visible light region and its high electrical conductivity [5,16,17,18,19,20,21,22]. Isolated Ag islands are formed on the substrate during the early nucleation growth of Ag film because of its Volmer–Weber growth mode, limiting the mean free path of the conduction electrons [14]. The percolation threshold thickness for an Ag film growing from isolated islands to a continuous layer is typically 10–20 nm [5,14], inevitably sacrificing the Ag film’s optical transparency and transparent band, which necessitates a modification of the growth mode to lower the percolation threshold of the Ag film. Techniques such as dielectric or metal wetting layer, metal doping, and molecular or polymer surfactant have been utilized to reduce the Ag layer’s percolation threshold [14]. 

TiO_2_/Ag/AZO and TiO_2_/Ag/ITO, with the wetting layer of TiO_2_ used to obtain an Ag film of 10 nm thick, have been proven to be useful transparent electrodes for polymer solar cells [17,23]. For solar cell applications, the transparent band of TC should always cover the visible-to-infrared light region to transmit most of the incident solar light. Nevertheless, for other applications, such as low-emission glass, it should only transmit visible light [24]. To obtain TCs with tunable transparent bands, the film structure of TiO_2_/Ag/AZO based on an ultrathin Ag layer was designed and fabricated, while the thickness of the Ag layer was considered as a tool with which to adjust the transparent spectral band. The optical and electrical properties of the as-deposited and annealed samples at different temperatures were carefully investigated. In addition, the flexibility of the samples was also studied in detail with different bending cycles and radii.

## 2. Experimental Section 

TiO_2_, Ag, and AZO (2 wt% Al_2_O_3_:ZnO) were deposited on K9 glass (1 mm), silicon (0.6 mm), and polyethylene terephthalate (PET, 188 μm) substrates by means of a magnetron sputtering system with high purity targets (>99.99%). Before the deposition process, the substrates were cleaned in acetone, alcohol, and deionized water sequentially in an ultrasonic bath, each process lasting 10 min, and dried by nitrogen flow. The background pressure was below 4.5 × 10^−6^ Torr, and with high-purity argon (Ar) injected into the chamber at 10 standard cubic centimeters per minute (SCCM), the growth pressure was controlled at 1 × 10^−3^ Torr by a throttle valve. In our work, the same growth pressure was employed to deposit all the layers. The vacuum was not broken during the deposition process of the entire structure. TiO_2_ and AZO were grown in radio-frequency (RF) mode with power at 100 W, while Ag was deposited in direct-current (DC) mode with power at 50 W. The substrate holder was rotated at 10 RPM (revolutions per minute). All the films were deposited without any intentional heating. The growth velocity and optical constants of each layer were characterized by a varied-angle spectroscopic ellipsometer at three incident angles of 65, 70, and 75° (ELLIP-SR-II, Shanghai Bright Enterprise Development Co., Shanghai, China).

Ar pressure, as an important process parameter for film deposition, has been studied and proven to influence the film structure of sputtering films, as well as the corresponding electrical, optical, and magnetic properties [25,26,27,28]. John A. Thornton proposed a model able to predict the structural forms of evaporated or sputtered materials depending on the two parameters of *T*/*T*_m_ (*T* is the substrate heating temperature and *T*_m_ is the melting point of the coating material) and internal gas pressure in the chamber [29]. Four structure zones existed in the sputtered films according to the characteristic structural forms. According to the model, the film structure was influenced by the Ar pressure. For DC-sputtering FeNi permalloy films, the Fe concentration and sharpness of the crystalline state in the films decreased with the increase in Ar pressure, while the surface roughness of the films exhibited a contrary variation trend [25]. The effects of Ar pressure on the structure and magnetic properties of sputter-deposited CoCrPt films were also studied [26]. The Ar pressure varied from 5.5 to 40 mTorr. Increasing the Ar pressure led to isolation enhancement and a size decrease of the CoCrPt grains. The coercivity increased with the Ar pressure up to 20–30 mTorr. In addition, the medium noise decreased significantly with the increase in Ar pressure. In the work proposed by Igasaki et al., the AZO films were deposited by RF sputtering, with the Ar pressure during deposition in the range of 0.08–2.7 Pa [27]. The results showed that the resistivity of the AZO film increased with the increase in Ar pressure, while its optical transmittance remained nearly unchanged. Similarly, the conductivity of the sputtered chromium thin films increased significantly as the Ar pressure grew lower [28]. Hence, an Ar pressure of 1 × 10^−3^ Torr (0.133 Pa) was chosen to obtain AZO and Ag films with high conductivity. Moreover, the value used in this work has been proven to deposit films with very smooth surface morphologies, which is beneficial for the purpose of obtaining TCs with low optical daze [30,31,32]. In addition, the low growth pressure accompanied by the lower sputtering power was adopted to control the growth rate of the ultrathin Ag layer. Hence, the ultrathin Ag layer can be deposited at a suitable time duration.

The samples were heat-treated in a tube furnace at temperatures of 200, 300, and 400 °C, respectively, in a vacuum of about 4 Pa. The temperature rising and cooling rates were both 10 °C/min, and the time at which the temperature was constant lasted 30 or 60 min.

The optical transmittance spectra of the samples were measured by a spectrophotometer, with air used for calibration (Lamda 950, PerkinElmer, Waltham, MA, USA). The sheet resistance was tested using the four-point probe technique (JG M-3 handheld Four-point probe tester, Suzhou Jingge Electronics Co., Ltd., Suzhou, China). Surface morphologies were obtained by atomic force microscopy (FSM-Nanoview 1000 AFM, Fishman Suzhou, Suzhou, China) in the tapping mode with a measured area of 10 × 10 μm^2^. Transmission electron microscopy was adopted to characterize the cross-sectional micrographs of the samples (TEM, Tecnai G2 F20, FEI, Hillsboro, OR, USA). A homemade one-dimensional motorized translation stage was used to characterize the flexibility of the samples on PET substrates at different bending cycles and radii.

## 3. Results and Discussion

### 3.1. Numerical Design and Simulation

For DMD-based TC, its optical loss can be greatly suppressed by means of antireflection design and employing ultrathin and low optical-loss metal layers [2]. Although the same material was always used for the undercoat and overcoat dielectric layers in most previous works, different materials have been suggested in order to obtain optimal broadband transparency in a realistic DMD structure [1,24]. Moreover, the undercoat dielectric layer should have a high refraction index, such as ZnO and TiO_2_, to achieve high visible transmittance [1,14]. According to the design rule, TiO_2_ was chosen as the undercoat dielectric layer, which can also serve as the wetting layer to obtain a continuous sub-10 nm Ag layer [1,2,17,21,23]. For the top dielectric layer, AZO was selected. The resistance of a DMD-based TC can be expressed as a function of parallel-connected resistance of the three individual layers, which is mainly determined by the metal layer because of the much lower resistivity of metal than that of the dielectric layer (~10^7^ Ω.cm and 10^−6^~10^−4^ Ω.cm for dielectric and metal layer, respectively) [21,33]. Nevertheless, if the dielectric layer were replaced by ITO or AZO, with resistivity comparable to that of the metal layer (10^−4^–10^−3^ Ω.cm [30,34,35]), the resistance of the DMD-based TC could be reduced further to some extent [2,17,23].

For the proposed three-layer TiO_2_/Ag/AZO film structure, the transmittance spectra can be numerically simulated by using the transfer matrix method (TMM), with optical constants of TiO_2_, Ag, and AZO measured in advance [36]. The characteristic matrix of a *q*-layered film system is described as

(1) $$\left[\begin{array}{c} B\\ C \end{array}\right]=\left\{\prod_{r=1}^{q}\left[\begin{array}{cc} \cos\phi_{r} & \frac{i}{\eta_{r}}\sin\phi_{r}\\ i\eta_{r}\sin\phi_{r} & \cos\phi_{r} \end{array}\right]\right\}\left[\begin{array}{c} 1\\ \eta_{m} \end{array}\right]$$

where *B* and *C* are the normalized electric and magnetic fields at the front interface.

The phase factor *ϕ* is defined as

(2) $$\phi_{r}=\frac{2\pi N_{r}d_{r}\cos\theta_{r}}{\lambda }$$

where *N*_r_ = *n*_r_ − i*k*_r_ is the complex refractive index of the *r*-th layer (*n* is the refractive index and *k* is the extinction coefficient), *d*_r_ is the thickness of the *r*-th layer, *θ*_r_ is the incident angle, and *λ* is the wavelength of the incident light. 

The optical admittance *η*_r_ of the *r*-th layer is given by

(3) $$\eta_{r}=N_{r}\cos\theta_{r}\mathrm{for}\;\mathrm{TE}\;\mathrm{wave}$$

(4) $$\eta_{r}=\frac{N_{r}}{\cos\theta_{r}}\mathrm{for}\;\mathrm{TM}\;\mathrm{wave}$$

In Equation (1), *η*_m_ is the optical admittance of the substrate. 

Reflectance *R* and transmittance *T* of a multilayered structure can be calculated by

(5) $$R=(\frac{\eta_{0}B-C}{\eta_{0}B+C})\left(\frac{\eta_{0}B-C}{\eta_{0}B+C}\right)*$$

(6) T=(1−R)ηmRe(BC*)

where *η*_0_ = 1 is the optical admittance in free space and (.)* indicates a complex conjugate.

The optical constants of TiO_2_, AZO, and Ag were characterized by a spectroscopic ellipsometer. The ellipsometric parameters (*Ψ*, Δ) were measured in a wavelength range of 300–1100 nm at three incident angles of 65, 70, and 75°. In the ellipsometric parameter fitting process, to characterize the optical dispersion relation of AZO and Ag films, the Lorentz–Drude model with three oscillators was employed [37]:

(7) $$\varepsilon =\varepsilon_{\infty }-\frac{E_{p}^{2}}{E(E+i\Gamma_{p})}+\sum_{j=1}^{3}\frac{A_{j}^{2}}{E_{j}^{2}-E^{2}+iE\Gamma_{j}}$$

where *ε*, *ε*_∞_, *E_p_*, and Γ*_p_* are the complex dielectric function, the high-frequency dielectric constant, the plasma frequency, and the Drude broadening factor, respectively. *A_j_*, *E_j_*, and Γ*_j_* are the oscillator strength, energy, and damping factor, respectively. 

For TiO_2_, the Cauchy exponential dispersion model was used [38]:

(8) $$n(\lambda )=A_{n}+\frac{10^{6}B_{n}}{\lambda^{2}}+\frac{10^{12}C_{n}}{\lambda^{4}}$$

(9) $$k(\lambda )=A^{\prime }e^{\left[B^{\prime }(\frac{1239.8}{\lambda }-{\;E}_{g})\right]}$$

where *λ* is in the unit of “nm”. *A_n_*, *B_n_*, *C_n_*, *A*′, *B*′, and *E*_g_ are the fitting parameters. 

The three-phase model consisting of Si/(TiO_2_ or AZO or Ag)/Air was used to extract the realistic film structure of the samples for ellipsometry analysis. Because of the thickness-dependent optical constants of the thin metal film with a thickness lower than its electron mean free path [37], a single-layer Ag film on silicon with a thickness of about 12 nm was fabricated and measured to obtain the optical constants of the ultrathin Ag layer, which would make the numerical design more accurate in terms of simulating the optical properties of the fabricated sample. 

**Figure 1:**
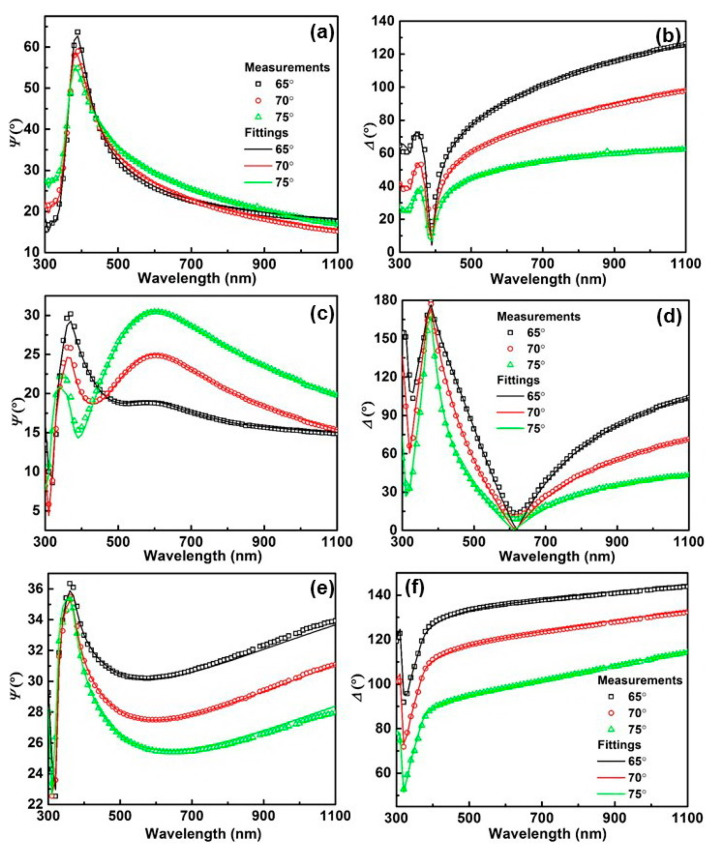
Measured and fitted ellipsometric parameters (*Ψ* and Δ) of (**a**,**b**) AZO, (**c**,**d**) TiO_2_, and (**e**,**f**) Ag films.

The measured and fitted ellipsometric parameters for the AZO, TiO_2_, and Ag films are given in Figure 1. To characterize the difference between the measured and fitted ellipsometric parameters, the parameter of the root mean square error (RMSE) is defined [37]:

(10) $$\mathrm{RMSE}=\sqrt{\frac{1}{2N-M}\sum_{i=1}^{N}\left[{\left(\frac{\Psi_{i}^{mod}-\Psi_{i}^{exp}}{\sigma_{\Psi ,i}^{exp}}\right)}^{2}+{\left(\frac{\Delta_{i}^{mod}-\Delta_{i}^{exp}}{\sigma_{\Delta ,i}^{exp}}\right)}^{2}\right]}$$

where *N* is the number of the measured points, *M* is the number of fitting parameters, and *σ* is the standard deviation. The superscripts mod and exp refer to the modeled and experimental data, respectively. The RMSE values for the fitting of TiO_2_, AZO, and Ag were 0.685, 0.930, and 0.234, respectively. The rather low RMSE values in accompaniment with the good agreement between the experimental and fitting curves, as depicted in Figure 1, demonstrates the accuracy and reliability of the fittings. 

**Figure 2:**
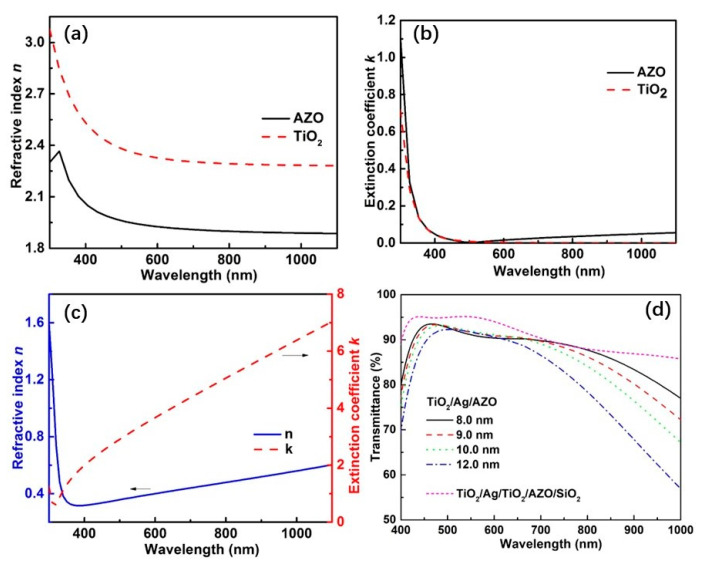
(**a**) Refractive index of AZO and TiO_2_, (**b**) extinction coefficient of AZO and TiO_2_, (**c**) refractive index and extinction coefficient of Ag ultrathin layer. (**d**) Simulated transmittance spectra of TiO_2_ (35.0 nm)/Ag (8.0/9.0/10.0/12.0 nm)/AZO (50.0 nm) and TiO_2_ (35.2 nm)/Ag (8.0 nm)/TiO_2_ (42.0 nm)/AZO (0.6 nm)/SiO_2_ (90.2 nm).

The refractive index *n* and extinction coefficient *k* of TiO_2_, AZO, and Ag are presented in Figure 2a–c. To obtain the highest visible optical transmittance of the proposed three-layer TC, the thicknesses of TiO_2_ and AZO were varied to fit the 100% optical transmittance in the wavelength range from 400 to 700 nm. In the optimization, the Ag layer’s thickness was fixed at 8 nm, which is the lowest thickness with which Ag was able to form a continuous film on the TiO_2_ wetting layer [23]. The resulting film’s structure was TiO_2_ (35.0 nm)/Ag (8.0 nm)/AZO (50.0 nm). Figure 2d shows the simulated transmittance spectra of the designed film structure, with varied film thicknesses for the Ag layer. As shown, the transparent band became narrow and the average transmittance decreased with the increase in the Ag layer’s thickness. 

In addition to the three-layered TiO_2_/Ag/AZO film structure, we also experimented with adding more dielectric layers to improve the optical transmittance in a broader wavelength range. The six-layer film structure of TiO_2_/Ag/AZO/TiO_2_/AZO/SiO_2_ was proposed, and its transmittance in the wavelength range of 400–700 nm was maximized by varying the film thicknesses of TiO_2_, AZO, and SiO_2_. In the optimization process, the optical constants of SiO_2_ were obtained from the reference of Palik [39]. The optimized film structure was TiO_2_ (35.2 nm)/Ag (8.0 nm)/TiO_2_ (42.0 nm)/AZO (0.6 nm)/SiO_2_ (90.2 nm). The transmittance spectra of the five-layered film structure are depicted in Figure 2d. As can be seen, they show higher transmittance values than those of the three-layered TiO_2_/Ag/AZO in nearly the entire wavelength range of 400–1000 nm, indicating that adding layers is beneficial to the improvement of the optical transmittance. Nevertheless, in the optimized five-layer film structure, the thickness of the AZO layer was nearly zero. According to the above discussions [17,23], in consideration of the comparable resistivity of AZO with that of metals, the nearly zero film thickness of AZO was detrimental to the conductivity increment of the whole film structure. In general, this topic is interesting and can be particularly investigated in future work. 

### 3.2. Experimental Results

**Figure 3:**
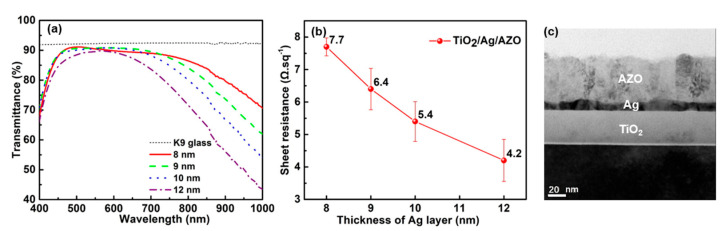
(**a**) Measured transmittance spectra of the samples based on different thicknesses of the Ag layer. The short dotted line represents the transmittance spectra of the K9 glass substrate. (**b**) The sheet resistance of the TC samples with different Ag layer thicknesses. (**c**) TEM image of the TC sample based on an Ag layer of 9 nm thickness.

The measured transmittance spectra of the samples with different Ag layer thicknesses are presented in Figure 3a. The average optical transmittance values in a range of 400–700 nm for the Ag-based TCs with thicknesses of 8.0, 9.0, and 10.0 nm were 88.3%, 88.9%, and 88.6%, respectively, and 86.2% for Ag-based TC 12.0 nm thick. As is clearly shown, the transparent band of the sample was tunable and shrunk with the increase in the Ag film’s thickness. Figure 3b gives the sheet resistance of TiO_2_/Ag/AZO with different thicknesses of the Ag layer. The sheet resistance decreased with the increase in the Ag film’s thickness, and the lowest resistance of 4.2 Ω.sq^−1^ was obtained for TC based Ag layer of 12.0 nm. Nonetheless, there were no radical differences in resistivity for any of the samples, implying that all of the Ag layers are continuous.

The cross-sectional TEM images of the samples based on an Ag layer of 9.0 nm thickness are depicted in Figure 3c, which clearly shows a tri-layer film structure with a continuous ultra-thin Ag layer. Although the layers of TiO_2_ and AZO were deposited at the same parameters, they exhibited rather different film structures. The TiO_2_ layer was dense and homogeneous, while the AZO layer showed a columnar structure. The same columnar structure was also found for the single AZO layer deposited on the Si substrate, as was illustrated in our previous work [30]. Hence, it is assumed that the columnar film structure of the AZO layer is mainly determined by its intrinsic growth mode. The ultrathin Ag layer with a thickness of about 9.0 nm showed a continuous structure benefiting from the undercoat TiO_2_ layer, verifying the wetting effect of the TiO_2_ layer [17]. In addition, the surface topography of the Ag layer influenced that of the overcoat AZO layer. Hence, it is believed that changing the deposition parameters, such as the Ar pressure, sputtering power, heating temperature, etc., can effectively tune the film structure and the optoelectrical properties of the proposed film. From the TEM image, the film thickness of each layer of the sample can be estimated to be about TiO_2_ (36.8 nm)/Ag (9.0 nm)/AZO (50.0 nm), which is in good agreement with the design.

**Figure 4:**
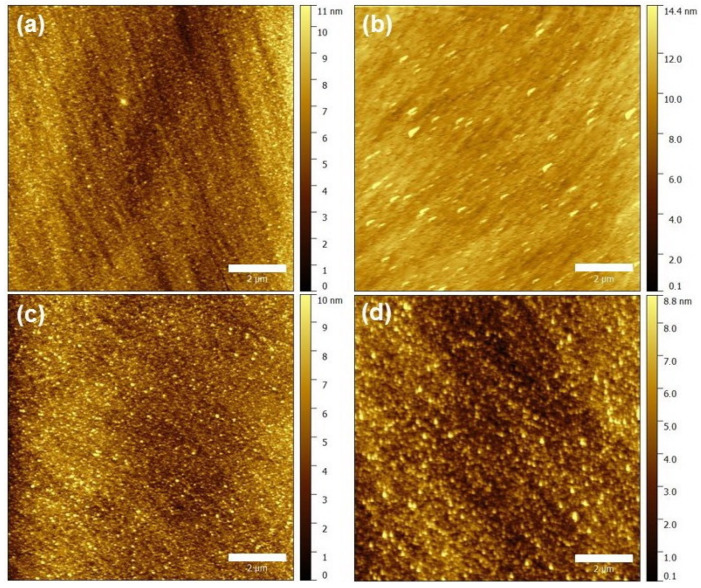
AFM micrographs of the TC sample with Ag layer thicknesses of (**a**) 8.0 nm, (**b**) 9.0 nm, (**c**) 10.0 nm, and (**d**) 12.0 nm. The scale bar is 2 μm.

Figure 4 gives the AFM images of the fabricated TC samples. All samples exhibited quite smooth surfaces, with surface roughness values of about 1.4 nm, indicating a low optical daze of the proposed TCs.

A figure-of-merit (FOM) with which to compare the performances of the proposed TCs based on different film thicknesses of the Ag layer was defined by Haacke, given by [40]:

(11) $$\mathrm{FOM}=\frac{T^{10}}{R_{s}}$$

Here, we used the average optical transmittance of 400–700 nm as *T* to characterize the average optical transparency in the visible light region (*T*_average_), rather than to consider only the transmittance at 550 nm [17]. *R*_s_ is the measured sheet resistance. The results are given in Table 1. Among the Ag layer-based TCs with thicknesses of 8.0, 9.0, 10.0, and 12.0 nm, the sample based on the 10.0 nm Ag layer showed the highest performance.

**Figure 5:**
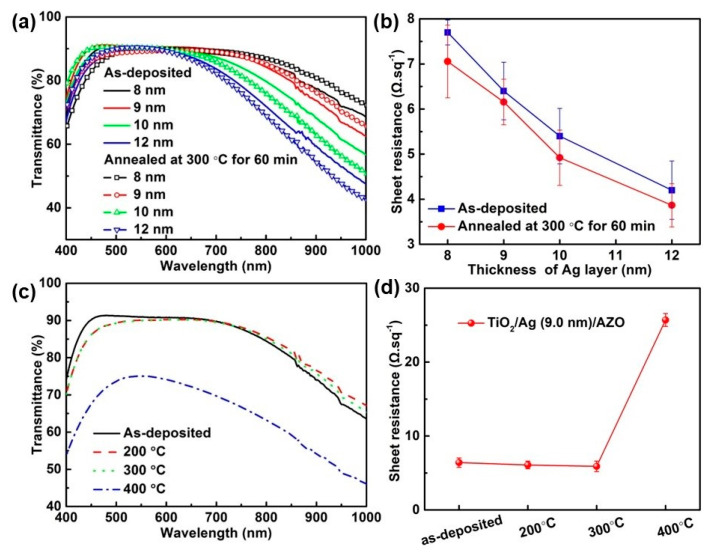
(**a**) Transmittance spectra and (**b**) sheet resistance of the as-deposited and annealed samples at 300 °C for 60 min. (**c**) Transmittance spectra and (**d**) sheet resistance of the Ag-based TC with 9.0 nm thickness after annealing at 200, 300, and 400 °C for 30 min.

The optical transmittance spectra and sheet resistance of the as-deposited and annealed samples after heat treatment at 300 °C for 60 min are depicted in Figure 5a,b. As can be seen, the transmittance was nearly unchanged after annealing for all the samples. Moreover, the sheet resistance of the annealed sample decreased compared with the as-deposited sample due to the improved crystallization and the reduction in defects and grain boundaries [41,42]. The TC sample based on an Ag layer 9.0 nm thick was also annealed at 200, 300, and 400 °C for 30 min, and the results are shown in Figure 5c,d. There was little change in the transmittance spectra of the sample after annealing at 200 and 300 °C, but a dramatic drop for the sample annealed at 400 °C, demonstrating the high-temperature application potential up to 300 °C. The sheet resistance of the sample decreased with an annealing temperature up to 300 °C. Nevertheless, after heat treatment at 400 °C for 30 min, the sheet resistance of the sample showed an obvious increase from 5.9 to 25.7 Ω.sq^−1^, which may be due to the oxidation of and damage to the continuity of the Ag layer caused by the TiO_2_ layer’s crystallization [13,43,44].

**Figure 6:**
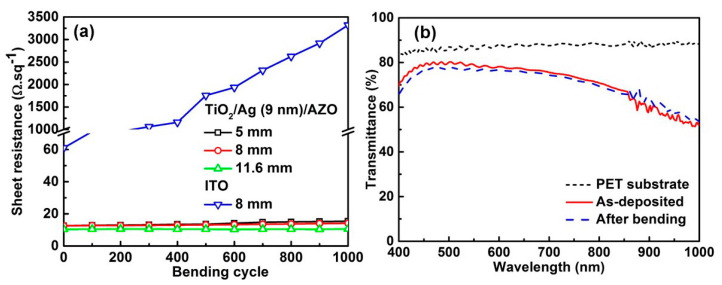
(**a**) Sheet resistance of the TC samples at different bending cycles and curvature radii. (**b**) Transmittance spectra of the sample based on the Ag layer with 9.0 nm thickness before and after 1000 bending cycles.

To verify the flexibility, the samples, on polymetric PET substrate, were subjected to a bending test using a homemade one-dimensional motorized translation stage. Here, the samples based on the Ag layer with 9.0 nm thickness were chosen. An ITO film with a thickness of about 100.0 nm was also tested as a contrast. The samples were repeatedly bent with different bending radii from 5 to 11.6 mm. Figure 6a gives the sheet resistance values of the samples after bending at radii of 5, 8, and 11.6 mm, respectively. The fabricated TC showed excellent mechanical flexibility compared with ITO due to the good mechanical ductility of the Ag film. After 1000 bending cycles, the change in *R*_s_ was smaller than 20%, even for the sample with the smallest curvature radius of 5 mm [2]. The transmittance spectra were also nearly unchanged after 1000 bending cycles, as shown in Figure 6b. The lower transmittance of the sample on the PET substrate than that of the sample on the K9 glass substrate may be due to the rough surface of the PET substrate, which needs to be modified. The deposition parameters of the TCs should also be optimized [45,46,47].

## 4. Conclusions

ITO-free transparent conductors based on three-layer TiO_2_ (35.0 nm)/Ag (8.0/9.0/10.0/12.0 nm)/AZO (50.0 nm) were designed and fabricated by means of magnetron sputtering. Benefiting from the favorable nucleation and wetting effect of the TiO_2_ layer and the low resistivity of the AZO layer, TCs with high optical transparency and electrical conductivity were obtained. By controlling the deposition process of the ultrathin Ag layer, the transparent band and FOM of the TC samples were adjusted. The TC-8 sample with the thinnest Ag layer exhibited an average optical transmittance of 88.3% (400–700 nm) and sheet resistance of 7.7 Ω.sq^−1^. Further increasing the Ag layer’s thickness to 10.0 nm retained the high optical transparency of the sample, but decreased the sheet resistance to 5.4 Ω.sq^−1^. After that, the optical transmittance dropped noticeably for TC-12. The highest FOM of 54.9 × 10^−3^ Ω^−1^ was obtained for the TC-10 sample. All the samples were able to hold their optical and electrical properties after annealing at 300 °C for 60 min, but failed at 400 °C, even at 30 min. Mechanical flexibility testing showed that the TC sample had less than a 20% relative change in sheet resistance after 1000 bending cycles at a curvature radius of 5 mm, exhibiting far higher mechanical robustness than the ITO film. These results demonstrate the potential of the proposed TCs for bendable and wearable optoelectronic devices requiring different transparent bands.

## Figures and Tables

**Table 1:** Performance comparison of the fabricated TCs based on different thicknesses of the Ag layer.

Sample	Thickness of Ag Layer (nm)	*T*_average_ (%)	*R*_s_ (Ω.sq^−1^)	Haacke FOM (×10^−3^ Ω^−1^)	Surface Roughness (nm)
TC-8	8.0	88.3	7.7	37.6	1.5
TC-9	9.0	88.9	6.4	48.2	1.0
TC-10	10.0	88.6	5.4	54.9	1.4
TC-12	12.0	86.2	4.2	53.9	1.4

## Data Availability

The data presented in this study are available on request from the corresponding author.
